# Periodic fluctuations in reading times reflect multi-word-chunking

**DOI:** 10.1038/s41598-023-45536-y

**Published:** 2023-10-28

**Authors:** Chia-Wen Lo, Mark Anderson, Lena Henke, Lars Meyer

**Affiliations:** 1 Research Group Language Cycles, Max Planck Institute for Human Cognitive and Brain Sciences, 04013 Leipzig, Germany; 2 Norwegian Computing Center, 0314 Oslo, Norway; 3 Clinic for Phoniatrics and Pedaudiology, University Clinic Münster, 48149 Münster, Germany

**Keywords:** Human behaviour, Language

## Abstract

Memory is fleeting. To avoid information loss, humans need to recode verbal stimuli into chunks of limited duration, each containing multiple words. Chunk duration may also be limited neurally by the wavelength of periodic brain activity, so-called neural oscillations. While both cognitive and neural constraints predict some degree of behavioral regularity in processing, this remains to be shown. Our analysis of self-paced reading data from 181 participants reveals periodic patterns at a frequency of $\sim$ 2 Hz. We defined multi-word chunks by using a computational formalization based on dependency annotations and part-of-speech tags. Potential chunk outputs were first generated from the computational formalization and the final chunk outputs were selected based on normalized pointwise mutual information. We show that behavioral periodicity is time-aligned to multi-word chunks, suggesting that the multi-word chunks generated from local dependency clusters may minimize memory demands. This is the first evidence that sentence processing behavior is periodic, consistent with a role of both memory constraints and endogenous electrophysiological rhythms in the formation of chunks during language comprehension.

## Introduction

Language comprehension has its limits: In order to understand speech, we must link words together—yet we cannot do so across sequences of arbitrary duration because memory contents progressively deteriorate with time^1,2^. Earlier work has shown that temporal integration of events into larger units is restricted to a window of 3 s^3^. For instance, performance on sequence reproduction tasks is high for sequences of up to 3 s^4^. Accordingly, electroencephalography research has observed that the contingent negative variation, a potential associated with the anticipation of events (such as their expectancy or duration), accompanies the reproduction of stimuli of 1–3 s, but reduces for intervals beyond 4 s^5^. Auditory short-term memory is limited to a similar interval of 2–3 s^6^. For language, a proposed window of 6 words^7^ translates to 2.4 s when assuming a rate of 150 words per minute^8^. Likewise, the duration of single utterances in speech approaches a median of 2.6 s^9^ (see also^10^).

More recently, it has been suggested that the pace of electrophysiological activity could explain such timing constraints. In particular, cycles of slow-frequency activity play a role in the cognitive formation of multi-word units. A seminal study presented native speakers of Mandarin and an English-speaking control group with isochronous 4-syllable sentences while recording their magnetoencephalogram^11^. Two syllables would always form a two-word phrase. Two phrases would always form a four-word sentence. The authors report spectral peaks at the rates of phrases (2 Hz) and sentences (1 Hz) in native speakers only, suggesting that the peaks reflect the formation of cognitive units during comprehension (see also^12^). In natural non-isochronous stimuli, phase angles of oscillatory activity in the delta band (< 4 Hz) predict the offsets of multi-word chunks^13^, in particular when chunks last for 2.7 s^14^ independent of acoustic boundary markings. Beyond speech, delta-band tracking was also reported for visual processing of sign language^15^ and lip movements^16^. This suggests that neural activity might impose an endogenous rhythm onto processing across domains.

Regardless of whether memory limitations or the wavelength of periodic activity are behind timing constraint on multi-word chunking, both would predict that behavioral data recorded during sentence comprehension should contain regular temporal patterns—although the text input does not contain any temporal markings. In particular, periodic behavioral events should align with chunk boundaries. This alignment may be reflected in the wrap-up effects, which are reading slowdowns that occur at the endings of clauses^17,18^ and implicit prosodic phrases^19^, implying that they reflect the cognitive formation of multi-word units^20^. Indeed, it was recently shown that eye movements during naturalistic sentence reading exhibit rhythmicity around 1 Hz that show coherence with the electroencephalogram^21^. However, naturalistic reading allows for word skipping and backward regressions^22^. It is, therefore, challenging to relate the observed periodicity to cognitive units and its behavioral relevance for language processing remains obscure.

**Figure 1:**
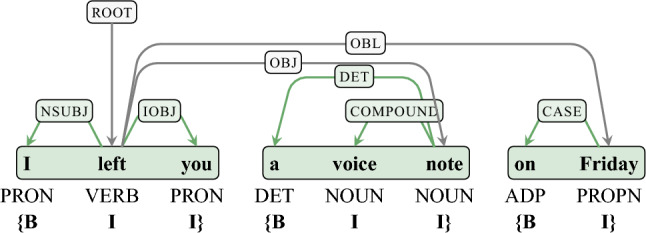
Formalization of chunks: chunks (green boxes, bottom) are automatically extracted based on the dependency relations and part-of-speech tags of a given sentence. Sentence examples are from UD English EWT treebank.

In the current study, we report for the first time that self-paced reading (SPR) data indeed also contain periodic patterns at a frequency that is consistent with periodic neuronal processes previously associated with chunking. Specifically, we show that these patterns align with chunk boundaries defined by our computational formalization. There are fruitful approaches for defining multi-word chunks through computational formalization. Different methods adopted for word tagging in an information extraction system might generate inconsistent output chunks^23,24^. Here we define multi-word chunks independently by using a computational formalism based on dependency annotations^25–27^, combining with the classic approach of a word-tagging system with the tagset of bio, where b means *the beginning of a chunk*, i means *inside a chunk*, and o means *outside of a chunk*^28^. This approach yields chunks that for the most part align with major syntactic boundaries—as exemplified in Fig. 1 (see Chunking Algorithm for details). These linguistically grounded chunk boundaries are established by finding the optimal set of sub-trees in a dependency tree. Often sentences can be chunked in more than one way, so we use an information theory process based on dependency relations and part-of-speech tags to prioritize more likely chunk candidates. Fundamentally, this means that the more often part-of-speech tags are connected to one another via specific dependency relations in a corpus, the more likely they will form a chunk in a given sentence.

Our findings provide the first behavioral evidence that reading behavior is regular at a slow time scale, consistent with both memory constraints on multi-word chunking and an involvement of rhythmic electrophysiological processes in the generation of multi-word chunks. Particularly, this periodic behavior seems to be relevant for the cognitive formation of multi-word units during higher-level language processing and may minimize memory demands.

## Results

### SPR times are periodic < 4 Hz

**Figure 2:**
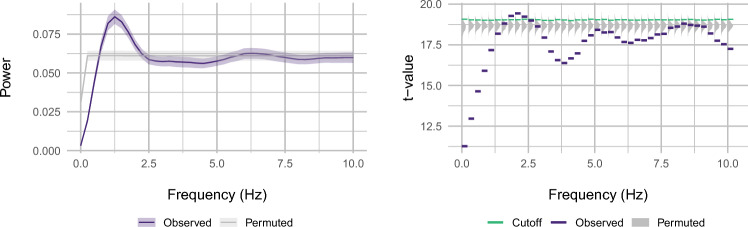
Results of spectral analysis: (**A**) power spectrum of differenced vector of log-transformed reading times (mean ± standard error); (**B**) test statistics from one-sample t-test within frequency bands of 0.25 Hz width; green lines mark 95th percentile of permutation distribution; both panels: purple = observed data, gray = permuted data.

We applied frequency-domain time-series analysis to wrap-up effects^29^ in N = 181 openly accessible SPR data sets^30^. To highlight wrap-up effects, we differenced the raw SPR time series, effectively amplifying transitions from slowdown to speedup across subsequent words (see Fig. 8B). For statistical analysis, we took a permutation approach (see Data Analysis). This revealed a peak around 2 Hz (see Fig. 2). Specifically, at 1.75, 2, and 2.25 Hz, the *t*-value of the one-sample *t*-test on the observed power estimates exceeded the 950th entry of the sorted distribution of *t*-values from tests on 1000 PSD spectra resulting from permutations of the differenced data, corresponding to an uncorrected one-tailed $p < 0.05$. After Bonferroni-correction for the 100 query frequencies, this remained significant ($p < 0.001$, corrected) at 2 Hz. These results suggest that natural, unconstrained reading slows down and then speeds up at a period of 0.5 s.

### Periodicity relates to chunking

To obtain chunks, we employ a computational model that defines them as sequences of words and bound morphemes that allow for all local dependencies to be established^25^ (for an example, see Fig. 7). The recognition of a chunk boundary has been shown psychologically real through different experimental paradigms (e.g. a click paradigm that has participants listen to sentences with clicks and indicate where the clicks are^31–33^) and analysis techniques (e.g. a hierarchical clustering scheme that data can be grouped by the measures of relatedness and then map them onto the hierarchical structure^34,35^). The approach we adopted here is analogous to formal linguistic definitions^26^ and resonates with classical phrase-structural approaches to chunking^27^. Note that the locality of these chunks implicitly minimizes memory demands, which are widely viewed as a key constraint on dependency processing^36^.

**Figure 3:**
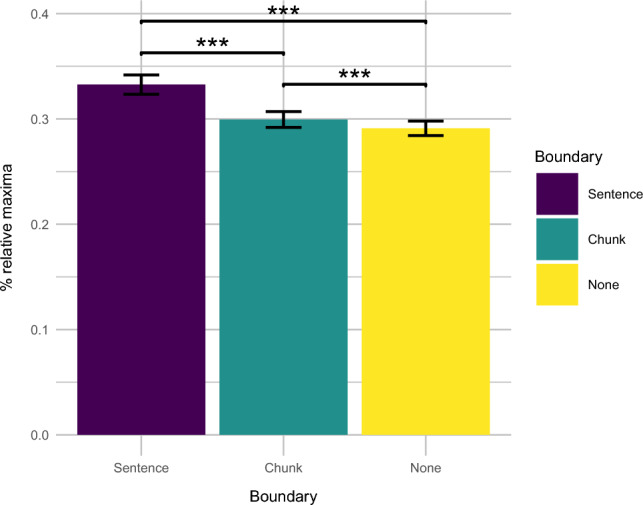
Percentage of relative maxima of differenced vector of log-transformed reading times (mean ± standard error) at sentence endings (purple), chunk endings (green), and non-boundary words (yellow); asterisks mark statistical significance at $p < 0.001$ in paired-samples *t*-tests.

To link periodic slowdown-speedup transitions to chunking, we first detected positive peaks in the differenced time series. These differences mark major transitions from slow to fast reading times. We then performed mixed-effects logistic regression analyses to assess whether the occurrence of a turning point depended on the presence of a chunk boundary. To stay consistent with prior literature on wrap-up effects, our boundary factor included not only a level for chunk boundaries, but also a level sentence for sentence boundaries; for comparison, a level non-boundary marked words that did not occur at either type of boundary. We first fitted a baseline model including an intercept, fixed effects of word frequency and word form surprisal, and random effects of subject and story. The baseline model was then compared to a model adding the boundary factor. Inclusion significantly improved model fit above baseline ($\chi ^{2}(2) = 588.82, p~<~0.001$). Analogous comparisons for subsets revealed significant model improvement within all condition pairs (sentence and chunk: $\chi ^{2}(1) = 371.30, p~<~001$; sentence and non-boundary: $\chi ^{2}(1) = 597.01, p < 0.001$; chunk and non-boundary: $\chi ^{2}(1) = 19.18, p < 0.001$; Fig. 3). This means that the transitions from slow to fast reading times occurred more often at sentence boundaries relative to both chunk boundaries and non-boundary words, and more often for chunk boundaries relative to non-boundary words.

### SPR slows down within chunks

**Figure 4:**
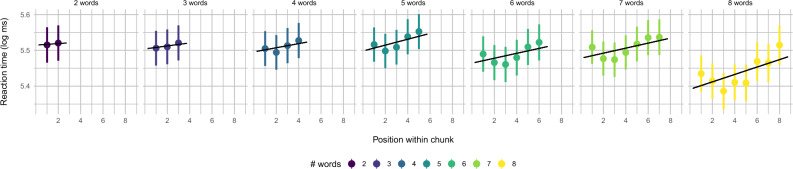
Reading times as a function of position within chunk; major abscissa/color shows chunk length increasing from left to right, minor abscissa show word position within chunk, the ordinate shows log-transformed reading times (mean ± standard error); regression lines mark means from within-chunk linear regression of log-transformed reading times on word position.

To substantiate the relevance of slowdown-speedups for chunking, we further assessed the progression of reading times within chunks, with the hypothesis that reading times increase gradually as readers approach the end of a chunk. To this end, within each chunk, we fitted a linear model that predicted reading time from word position. We then extracted the slope for each chunk and entered all slopes as a dependent measure into a new model with an intercept only, plus random factors for subject and story. There was a significant positive effect of the model intercept ($t(11.98) = 4.34, p < 0.001$, Satterthwaite-approximated Degrees of Freedom; Fig. 4). This suggests that reading times increase across word positions within chunk.

## Discussion

Our analyses provide the first behavioral evidence that higher-level language comprehension—specifically, the formation of multi-word memory chunks—is a periodic behavior. Previous work in cognitive neuroscience has linked slow periodic neural activity to eye movements during reading^21^ and the formation of multi-word chunks during language comprehension^11,13,14^. The current results suggest that this is indeed behaviorally relevant for language processing. Readers slow down and then speed up roughly every 0.5 s—mostly at sentence and chunk boundaries. They also show a gradual increase in SPR times from chunk onset to offset, which may indicate the incremental integration of words into a multi-word unit that progressively increases in size, consistent with neurophysiological evidence for a gradual increase of electrophysiological activity towards the end of each multi-word unit within a sentence^37^. Periodicity may thus reflect chunking. Classical wrap-up effects could reflect the periodic formation of multi-word chunks^29^.

The tendency of SPR transitions to occur at the boundaries of sentences and chunks links rather well to prior psycholinguistic proposals. In particular, it has been argued that memory constraints limit the distance of dependencies between the words and bound morphemes of sentences^36,38^. Here, we provide a complimentary hypothesis inspired by electrophysiology: If the wavelength of neural oscillations limits the duration of chunks, it would implicitly enforce short dependency distances to allow for dependency formation within the current memory chunk^1,27^. Nevertheless, the current data cannot dissociate this syntactic approach from perceptual notions of chunking. Likely, many of the chunk boundaries as defined here align with implicit prosodic boundaries. In spoken language, there is a strong alignment between syntactic and prosodic boundaries^39–41^. In the absence of prosodic markings, both listeners and readers generate implicit prosodic structure to guide perceptual sampling^42^. Moreover, implicit prosody is also reflected in periodic brain activity at delta-band frequency^43^. We embrace the classical view that perceptual sampling in time windows that cover multiple words and the formation of fine-grained dependency structure amongst these words go hand in hand^7^. Future research needs to investigate how such a staged architecture maps onto periodic neural and cognitive processes.

The current findings could provide an initial hint at a possible relationship between periodic slowdowns in reading and the periodicity of the electrophysiology of chunking. M/EEG studies have argued that delta-band oscillations (< 4 Hz) reflect the grouping of words into larger units^11,13,14^. Consistent with this, we observe a spectral peak at 2 Hz in reading times. Strikingly, the SPR data analyzed here do not contain any physical rhythm or boundaries. As chunk boundaries are not marked visually, they must be set by some cognitive heuristic^31,44^. Yet, given that the current study did not assess concurrent M/EEG in addition to behavioral responses, it remains to be shown that behavioral periodicity indeed stems from endogenous neuronal rhythms that synchronize with higher-level linguistic information^45–47^.

The current chunking formalism operates within the framework of dependency grammar^48^, which does not explicitly assume a hierarchical syntactic structure. Different types of cognitive units above the single-word level have been linked to periodic brain activity (for discussion, see^49–51^—some hierarchical, some not^11,13,14^). In principle, from the current results, we may only claim that the size of chunks may relate to the size of a neural processing window in the delta-band. The microstructure of syntax and syntactic processing within chunks is beyond the scope of the current work.

## Conclusion

Readers speed up and slow down periodically at a period of 0.5 s. These transitions may indicate the formation of multi-word chunks that allow for establishing all dependencies amongst the words and bound morphemes held in working memory at a time. Multi-word chunking is a periodic behavior, possibly mirroring underlying rhythmic neuronal processes.

## Methods

### Data

We analyzed a set of openly-accessible self-paced reading (SPR) data from 181 native speakers of English^30^. Participants had been instructed to read 10 stories from the Natural Stories Corpus word by word, advancing through button press. The reading time was measured for each word from the presentation onset to the button-press. Each story includes roughly 1000 words, which results in 10,245 words and 485 sentences in total. The text was automatically parsed using the Stanford Parser^52^. The output from the parser was manually corrected and automatically converted to the annotations for the Universal Dependencies (UD) by Futrell et al.^30^, so the data has high-quality human-verified UD annotations.

### Chunking algorithm

The processes described below are applied to the human-verified UD annotations of the Natural Stories Corpus. The chunks require no generalization as they, and the statistics used to derive them, are drawn directly from the UD annotations. The algorithm fundamentally gives a solution to finding base-level subtrees when more than one solution exists.

We define chunks as sequences of words and bound morphemes that form saturated local dependency clusters^25–27^. The chunking algorithm employs dependency annotations and part-of-speech tags^48^. Specifically, chunks are considered base-level subtrees, allowing for a language-agnostic definition and annotation. This means the core algorithm is based on subtrees with a depth of 1. However, this restriction is softened to allow for chunks with a depth of 2 to minimize unitary chunks using a simple heuristic as described below.

As a first step, potential candidate chunks are extracted. For a given sentence and its corresponding tree, the span between each node *n* at position *x* and its corresponding head *h* at position *k* (where *k* can be greater than or less than *x*) is considered a candidate chunk if the nodes between position *x* and $k-1$ (if $k>x$) or between $k+1$ and *x* (if $k<x$) all have the same head *h*. This process results in potentially overlapping chunks (e.g., the head of one chunk could be a dependent in another). To select the optimal chunk annotation for a given tree, each chunk is scored based on normalized pointwise mutual information (NPMI)^53^. We use the NPMI between the Universal part-of-speech (UPOS) tag of a node (*t*) and the tuple of the UPOS of the head of that node (*ht*) and the relation between the node and its head (*rel*). Such that for a given node:

1 $${\text{PMI}}(t;ht,rel) = \log \frac{{p(t;ht,rel)}}{{p(t)p(ht,rel)}}$$

2 $$\begin{aligned}{} & {} \text {NPMI}(t;ht,rel) = \frac{\text {PMI}}{-log(p(t;ht,rel)} \end{aligned}$$

and the average NPMI of a potential chunk is:

3 $$\begin{aligned} \langle \text {NPMI}\rangle = \frac{1}{N-1}\sum _{d\in C} \text {NPMI}(t_d;ht_d,rel_d) \end{aligned}$$

where *N* is the number of nodes in a phrase and *d* is a dependent in a phrase *C*. The potential chunks in a given tree are then selected greedily. That is the potential chunks are ordered based on their NPMI and the highest is selected first resulting in any conflicting chunk annotations being removed. This is repeated until no potential chunk labels are left.

**Figure 5:**
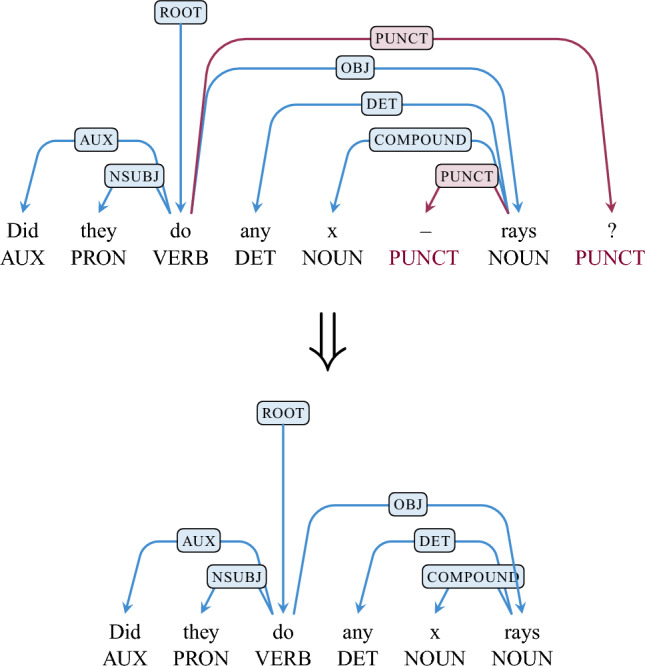
The top tree shows the original tree with two punctuation tokens. The nodes corresponding to tokens ‘?’ and ‘–’ (highlighted in magenta) are removed as they are tagged as PUNCT and neither have any dependents. The corresponding edges (also in magenta) are also cut from the tree. The resulting tree is shown below.

This process results in a large number of unitary chunks (i.e., chunks with only one node) which is unlikely to echo the multi-word units of natural language. In order to rectify this, two simple heuristics were applied. The first removes superfluous punctuation (superfluous with respect to the syntactic tree). Punctuation is only removed if a node has a UPOS tag of PUNCT *and* has no dependents. An example is shown in Fig. 5.

**Figure 6:**
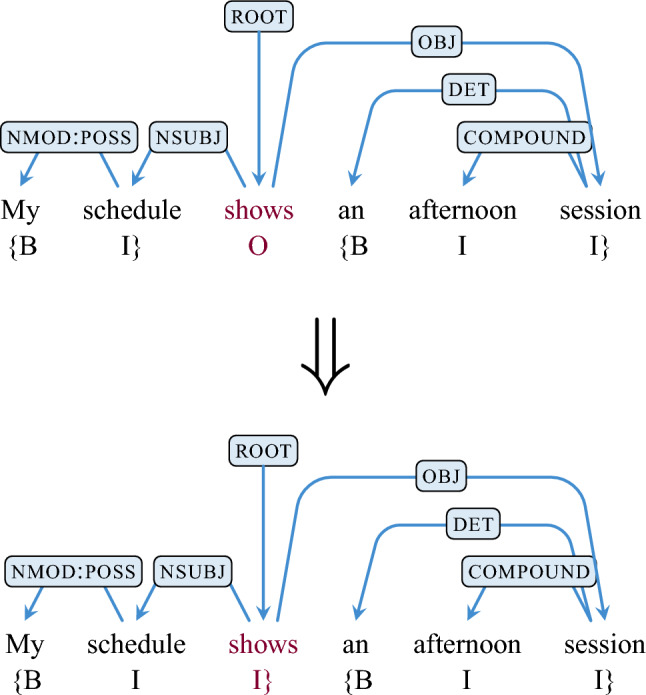
Labelling of chunks: **B**eginning of the chunk; **I**nside the chunk; **O**utside the chunk. The unitary chunk formed of the token *shows* (highlighted in magenta in the top tree) is labelled **O**. Possible chunks to attach it to are tried in order of their NPMI. This results in the unitary chunk being appended to the chunk consisting of *My* and *schedule*. The resulting chunk forms *My schedule shows*.

**Figure 7:**
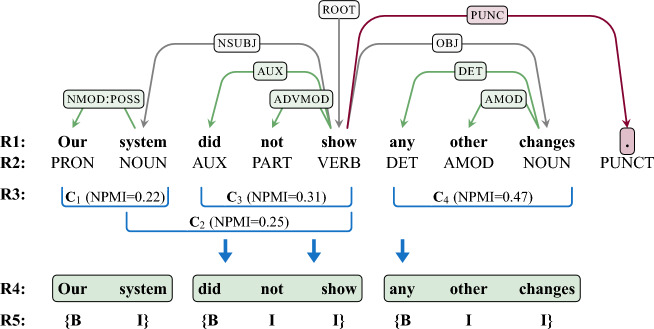
Applying the chunker algorithm to a UD dependency tree. **R1** shows the tokens of the sentence with the corresponding dependency tree above it. **R2** shows the corresponding part-of-speech tags for each token. Then candidate chunks are found based on the dependencies of each of the tokens, such that each candidate chunk has only one level of depth and is continuous. The candidate chunks are highlighted in **R3**. There is often more than one way to apply candidate chunks for a given sentence, as is the case in this example. The optimal combination is then selected based on the average NPMI of each candidate chunk which is calculated using the dependency relation types and part-of-speech tags of each token in a given chunk and on the corresponding statistics from the treebank. The candidate chunks are sorted by NPMI and then selected greedily with other candidate chunks being removed from the list if they conflict with the chosen chunk. In the example here, the candidate chunks would be sorted as: $C_4 (0.47), C_3 (0.31), C_2 (0.23),$ and $C_1 (0.22)$ with their NPMI values in parenthesis. So $C_4$ would be selected first, followed by $C_3$. Because $C_2$ is no longer viable as it overlaps with $C_3$, it is removed from the list of candidate chunks, leaving just $C_1$ which is then selected. The the full stop (period) is removed from the process as it has no dependencies (see Fig. 5). **R4** highlights the annotated chunks resulting from this process with **R5** showing the corresponding chunk labels.

The second heuristic attaches floating unitary nodes to chunks. This in effect removes the single depth restrictions of chunks, which was only introduced to simplify the original engineering use of this method. If a unitary chunk occurs at the boundary of a multi-token chunk and is syntactically linked to any element in that chunk (i.e., is the head or dependent of a node in the chunk), it is included in that chunk and the annotation is updated. Punctuation is treated slightly different. Any punctuation nodes that remain after applying the first heuristic is considered part of a chunk if it satisfies the boundary condition (with the syntactic criterion ignored), as the punctuation does not impact the analysis. An example is shown Fig. 6. Then the derived chunks are viewed as components, which can be a word or a sequence of words that takes into account inter-word relationships such as precedence and dominance. The overall process of generating chunk outputs is summarized in Fig. 7.

### Data analysis

**Figure 8:**
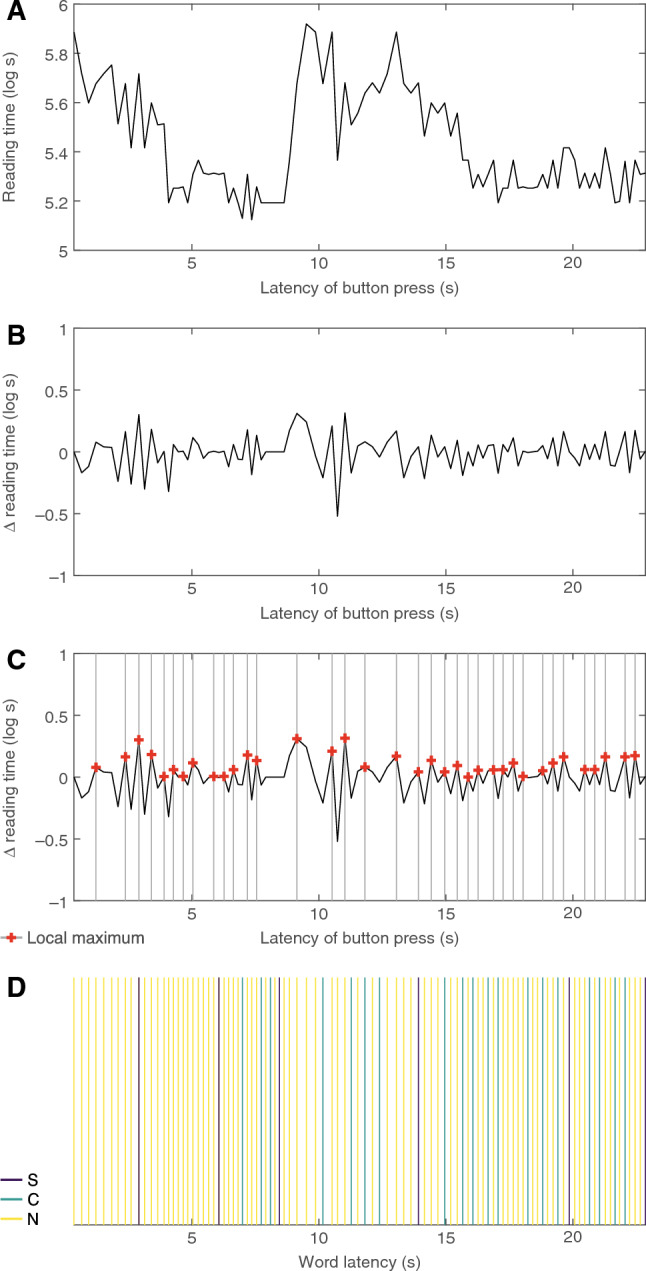
Data processing steps: (**A**) time series of log-transformed raw reading times from roughly 22 s of data from an example story and subject; each value is plotted at its corresponding latency relative to story onset; (**B**) differenced time series; (**C**) differenced time series with local maxima marked (red); (**D**) latencies of words at sentence boundaries (S; purple), chunk boundaries (C; green), and non-boundary words (N; yellow).

Preprocessing and spectral analysis were performed in MatLab^®^ (The MathWorks, Inc., US); statistical analysis was performed in R^54^. The authors of the original corpus suggested trimming reading times outside a range of 100–3000 ms^30^. Because the complete removal of word reading times would have disrupted the spectral analysis, and thereby, the actual pace of reading, we kept the original latency of each button press relative to story onset and data values outside the range of 100–3000 ms were replaced with a median value. The median was calculated within subject and story. Imputation affected 6 % of data values. Reading times were then log-transformed to achieve a normal distribution (Fig. 8A). For highlighting chunking-related slowdown–speedup transitions in the data, we performed differencing on the imputed vector of reading times (Fig. 8B). This decision was based on prior evidence for reading-time slowdowns at the end of clauses and sentences^55,56^ (for review, see^29^) and independent evidence from visual chunking in non-human primates that observed changes in reaction times at chunk boundaries^57^. In the differenced vector, local maxima reflect transitions from slowdowns to speedups between adjacent words (Fig. 8C). After differencing, data were converted to a time series sampled at 1000 Hz. The original latency of each button press in milliseconds relative to story onset served as index, the log-transformed reading time served as value.

The time series within subject and story then underwent short-term Fourier transform using Welsh’s power spectral density (PSD) estimation (window length = 4168 samples, overlap = 2084 sample, frequency resolution = 0.1 Hz); PSD was converted to power (see Fig. 2 for results). Statistical analysis employed a permutation approach: First, observed spectra were averaged within subject across stories and a one-sample *t*-test was performed across subjects within frequency bin; the *t*-statistic was noted. Second, a distribution of estimates for comparison was generated: Within story and subject, 1000 random time series were generated by randomly permuting the differenced values and inserting them at the observed indices. Spectra were averaged within permutation run and subject across stories; within run, a one-sample *t*-test was performed across subjects within frequency bin. Third, within frequency bin, we sorted the test statistics from the permuted data and assessed whether the observed statistic would surpass the $950^{\textrm{th}}$ value, corresponding to one-tailed $p~<~0.05$^58^, and then Bonferroni-corrected for the 100 query frequencies.

To relate slowdown–speedup transitions to chunking, we performed mixed-effects logistic regression analyses using the lme4 package^59^ in R. Words at sentence boundaries were defined as words followed by a period, question/exclamation mark, comma, or (semi)colon. Words at chunk boundaries were defined by the chunker. This means that sentence boundaries and chunk boundaries were mutually exclusive. Non-boundary words were all remaining words (Fig. 8D). Baseline and improvement models were compared using Analysis of Variance. Word frequency was determined with the wordfreq module in Python; word form surprisal was calculated using the minicons module in Python, based on GPT2^60^. Frequency and surprisal were included as nuisance regressors in all models because of their well-known influence on processing effort (for review, see^61,62^). Before inclusion, frequency and surprisal were scaled and centered.

As a second strategy for relating slowdown–speedup transition to chunking, we assessed the progression of reading times within chunks. We took a two-level approach: First, within chunk, we linearly regressed reading time on word position. Second, regression slopes (i.e., $\beta$ coefficients within chunk) were entered as dependent measure into a linear mixed model, fixed effect being only an intercept, random effects being subject, story, and chunk length (i.e., number of words within chunk); note that a random-slope model failed to converge.

## Data Availability

All data generated or analyzed during this study are included in Futrell et al.^30^.

## References

[1] Christiansen MH, Chater N (2016). The now-or-never bottleneck: A fundamental constraint on language. Behav. Brain Sci..

[2] Vasishth S, Nicenboim B, Engelmann F, Burchert F (2019). Computational models of retrieval processes in sentence processing. Trends Cogn. Sci..

[3] Pöppel E (1997). A hierarchical model of temporal perception. Trends Cogn. Sci..

[4] Pöppel, E. Oscillations as possible basis for time perception. In *The Study of Time* (eds Fraser, J. *et al.*) 565–589 (Springer, 1972).

[5] Elbert T, Ulrich R, Rockstroh B, Lutzenberger W (1991). The processing of temporal intervals reflected by CNV-like brain potentials. Psychophysiology.

[6] Baddeley AD, Thomson N, Buchanan M (1975). Word length and the structure of short-term memory. J. Verbal Learn. Verbal Behav..

[7] Frazier L, Fodor JD (1978). The sausage machine: A new two-stage parsing model. Cognition.

[8] Tauroza S, Allison D (1990). Speech rates in British English. Appl. Linguist..

[9] Vollrath M, Kazenwadel J, Krüger HP (1992). A universal constant in temporal segmentation of human speech. A reply to Schleidt and Feldhütter (1989). Naturwissenschaften.

[10] Vetchinnikova S, Konina A, Williams N, Mikušová N, Mauranen A (2023). Chunking up speech in real time: Linguistic predictors and cognitive constraints. Lang. Cogn..

[11] Ding N, Melloni L, Zhang H, Tian X, Poeppel D (2016). Cortical tracking of hierarchical linguistic structures in connected speech. Nat. Neurosci..

[12] Ding N (2017). Characterizing neural entrainment to hierarchical linguistic units using electroencephalography (EEG). Front. Hum. Neurosci..

[13] Meyer L, Henry MJ, Gaston P, Schmuck N, Friederici AD (2016). Linguistic bias modulates interpretation of speech via neural delta-band oscillations. Cereb. Cortex.

[14] Henke L, Meyer L (2021). Endogenous oscillations time-constrain linguistic segmentation: Cycling the garden path. Cereb. Cortex.

[15] Brookshire G, Lu J, Nusbaum HC, Goldin-Meadow S, Casasanto D (2017). Visual cortex entrains to sign language. Proc. Natl. Acad. Sci. U.S. A..

[16] Bourguignon M, Baart M, Kapnoula EC, Molinaro X (2020). Lip-reading enables the brain to synthesize auditory features of unknown silent speech. J. Neurosci..

[17] Just MA, Carpenter PA (1980). A theory of reading: From eye fixations to comprehension. Psychol. Rev..

[18] Rayner K, Kambe G, Duffy SA (2000). The effect of clause wrap-up on eye movements during reading. Q. J. Exp. Psychol..

[19] Hirotani M, Frazier L, Rayner K (2006). Punctuation and intonation effects on clause and sentence wrap-up: Evidence from eye movements. J. Mem. Lang..

[20] Clifton C, Carlson K, Frazier L (2002). Informative prosodic boundaries. Lang. Speech.

[21] Henke L, Lewis AG, Meyer L (2023). Fast and slow rhythms of naturalistic reading revealed by combined eye-tracking and electroencephalography. J. Neurosci..

[22] Rayner K (1998). Eye movements in reading and information processing: 20 years of research. Psychol. Bull..

[23] Nugues, P. M. *Language Processing with Perl and Prolog* 2nd edn. (Springer, 2014).

[24] Abney S (2007). Semisupervised Learning for Computational Linguistics.

[25] Anderson, M., Vilares, D. & Gómez-Rodríguez, C. Artificially evolved chunks for morphosyntactic analysis. In *Proceedings of the 18th International Workshop on Treebanks and Linguistic Theories (TLT, SyntaxFest 2019)* 133–143. 10.18653/v1/W19-7815 (Association for Computational Linguistics, Paris, France, 2019).

[26] de Marneffe M-C, Nivre J (2019). Dependency grammar. Annu. Rev. Linguist..

[27] Abney, S. Parsing by chunks. In *Principle-Based Parsing Studies in Linguistics and Philosophy* Vol. 44 (eds Berwick, R., Abney, S. & Tenny, C.) (Springer, 1991).

[28] Ramshaw, L. & Marcus, M. Text chunking using transformation-based learning. In *Third Workshop on Very Large Corpora* (1995).

[29] Stowe LA, Kaan E, Sabourin L, Taylor RC (2018). The sentence wrap-up dogma. Cognition.

[30] Futrell R (2021). The natural stories corpus: A reading-time corpus of English texts containing rare syntactic constructions. Lang. Resour. Eval..

[31] Fodor J, Bever T (1965). The psychological reality of linguistic segments. J. Verbal Learn. Verbal Behav..

[32] Holmes VM, Forster KI (1970). Detection of extraneous signals during sentence recognition. Percept. Psychophys..

[33] Holmes VM, Forster KI (1972). Click location and syntactic structure. Percept. Psychophys..

[34] Johnson SC (1967). Hierarchical clustering schemes. Psychometrika.

[35] Levelt W (1970). Hierarchial chunking in sentence processing. Percept. Psychophys..

[36] Futrell R, Mahowald K, Gibson E (2015). Large-scale evidence of dependency length minimization in 37 languages. Proc. Natl. Acad. Sci. U. S. A..

[37] Nelson MJ (2017). Neurophysiological dynamics of phrase-structure building during sentence processing. Proc. Natl. Acad. Sci. U. S. A..

[38] Petrini, S. & Ferrer-i-Cancho, R. The distribution of syntactic dependency distances. arXiv:2211.14620 (2022).

[39] Wagner M, Watson DG (2010). Experimental and theoretical advances in prosody: A review. Lang. Cogn. Process..

[40] Grosjean F, Grosjean L, Lane H (1979). The patterns of silence: Performance structures in sentence production. Cogn. Psychol..

[41] Truckenbrodt H (1999). On the relation between syntactic phrases and phonological phrases. Linguist. Inq..

[42] Breen M (2014). Empirical investigations of the role of implicit prosody in sentence processing. Lang. Linguist. Compass.

[43] Glushko A, Poeppel D, Steinhauer K (2022). Overt and implicit prosody contribute to neurophysiological responses previously attributed to grammatical processing. Sci. Rep..

[44] Buxó-Lugo A, Watson DG (2016). Evidence for the influence of syntax on prosodic parsing. J. Mem. Lang..

[45] Meyer L, Sun Y, Martin AE (2020). Synchronous, but not entrained: Exogenous and endogenous cortical rhythms of speech and language processing. Lang. Cognit. Neurosci..

[46] Rimmele JM, Morillon B, Poeppel D, Arnal LH (2018). Proactive sensing of periodic and aperiodic auditory patterns. Trends Cogn. Sci..

[47] Giraud A-L (2020). Oscillations for all$$^-\backslash$$\_ (-) \_/$$^-$$? a commentary on Meyer, Sun & Martin (2020). Lang. Cogn. Neurosci..

[48] Nivre, J. *et al.* Universal dependencies v1: A multilingual treebank collection. In *Proceedings of the Tenth International Conference on Language Resources and Evaluation (LREC’16)* 1659–1666 (2016).

[49] Kazanina N, Tavano A (2023). What neural oscillations can and cannot do for syntactic structure building. Nat. Rev. Neurosci..

[50] Lo C-W, Henke L, Martorell J, Meyer L (2023). When linguistic dogma rejects a neuroscientific hypothesis. Nat. Rev. Neurosci..

[51] Kazanina N, Tavano A (2023). Reply to ‘when linguistic dogma rejects a neuroscientific hypothesis’. Nat. Rev. Neurosci..

[52] Klein, D. & Manning, C. D. Accurate unlexicalized parsing. In *Proceedings of the 41st Annual Meeting of the Association for Computational Linguistics* 423–430. 10.3115/1075096.1075150 (Association for Computational Linguistics, Sapporo, Japan, 2003).

[53] Bouma, G. Normalized (pointwise) mutual information in collocation extraction. In *From Form to Meaning: Processing Texts Automatically, Proceedings of the Biennial GSCL Conference 2009* (2009).

[54] R Core Team. *R: A Language and Environment for Statistical Computing*. R Foundation for Statistical Computing, Vienna, Austria (2017).

[55] Mitchell DC, Green DW (1978). The effects of context and content on immediate processing in reading. Q. J. Exp. Psychol..

[56] Hill, R. L. & Murray, W. S. Commas and spaces: Effects of punctuation on eye movements and sentence parsing. In *Reading as a Perceptual Process* 565–589 (Elsevier, 2000).

[57] Tosatto L, Fagot J, Nemeth D, Rey A (2022). The evolution of chunks in sequence learning. Cogn. Sci..

[58] Maris E, Oostenveld R (2007). Nonparametric statistical testing of EEG-and MEG-data. J. Neurosci. Methods.

[59] Bates D, Mächler M, Bolker B, Walker S (2015). Fitting linear mixed-effects models using lme4. J. Stat. Softw..

[60] Radford A (2019). Language models are unsupervised multitask learners. OpenAI Blog.

[61] Hale J (2016). Information-theoretical complexity metrics. Lang. Linguist. Compass.

[62] Sassenhagen J (2019). How to analyse electrophysiological responses to naturalistic language with time-resolved multiple regression. Lang. Cogn. Neurosci..

